# Thresholding Approach for Low-Rank Correlation Matrix Based on MM Algorithm

**DOI:** 10.3390/e24050579

**Published:** 2022-04-20

**Authors:** Kensuke Tanioka, Yuki Furotani, Satoru Hiwa

**Affiliations:** 1 Department of Biomedical Sciences and Informatics, Doshisha University, Kyoto 610-0394, Japan; shiwa@mail.doshisha.ac.jp; 2 Graduate School of Life and Medical Sciences, Doshisha University, Kyoto 610-0394, Japan; yfurotani@mis.doshisha.ac.jp

**Keywords:** cross-validation, proportional threshold, sparse estimation

## Abstract

Background: Low-rank approximation is used to interpret the features of a correlation matrix using visualization tools; however, a low-rank approximation may result in an estimation that is far from zero, even if the corresponding original value is zero. In such a case, the results lead to a misinterpretation. Methods: To overcome this, we propose a novel approach to estimate a sparse low-rank correlation matrix based on threshold values. We introduce a new cross-validation function to tune the corresponding threshold values. To calculate the value of a function, the MM algorithm is used to estimate the sparse low-rank correlation matrix, and a grid search was performed to select the threshold values. Results: Through numerical simulation, we found that the false positive rate (FPR), interpretability, and average relative error of the proposed method were superior to those of the tandem approach. For the application of microarray gene expression, the FPRs of the proposed approach with d=2,3 and 5 were 0.128, 0.139, and 0.197, respectively, while the FPR of the tandem approach was 0.285. Conclusions: We propose a novel approach to estimate sparse low-rank correlation matrices. The advantage of the proposed method is that it provides results that are interpretable using a heatmap, thereby avoiding result misinterpretations. We demonstrated the superiority of the proposed method through both numerical simulations and real examples.

## 1. Background

It is essential in applications to compute the correlation matrix and present it with a heatmap [1] to understand the relationship between variables or subjects. However, when the sample size is small or contains noise, the correlation matrix may be challenging to interpret regarding the relationship between variables. Here, a correlation matrix that is easy to interpret is referred to as one that satisfies the following two properties: (a) the correlation coefficients between variables that are related are high and those that are not zero or close to zero, and (b) the number of correlation coefficients to be interpreted is small. In this paper, we deal with the problem of estimating such an easily interpretable correlation matrix.

A low-rank approximation address problem (a) [2]. Various methods have been proposed to estimate low-rank correlation matrices [3,4,5,6], and there are several advantages associated with this estimation. As one of these advantages, low-rank approximations can also effectively describe the clustering structure [7], which results in improved interpretations. These specific relations between variables are emphasized from the property, and therefore problem (a) can be solved.

While the low-rank correlation matrix has the above advantages, (i) the number of correlation coefficients to be interpreted becomes problematic when the number of variables is large, making interpretation difficult, and (ii) according to the Eckart–Young–Mirsky theorem [8], the error between the low-rank correlation matrix and the original correlation matrix becomes large, and even if the true correlation coefficient is 0, it can be estimated as a value far from 0.

To overcome this, in this study, we proposed a new approach to estimate sparse low-rank correlation matrices. The proposed approach, combined with a heatmap, provides a visual interpretation of the relationships between the variables. There are two types of techniques available for the sparse methods of the correlation matrix and the covariance matrix [9,10]. The first involves adding a sparsity penalty to the objective functions [11,12,13,14,15,16,17,18,19]. The other type uses thresholding values to achieve a sparse structure. Bickel and Levina (2008) proposed the thresholding matrix estimator [20] and various related methods have been developed [21,22,23,24]. In addition, to estimate the sparse correlation matrix, Refs. [25,26,27] used generalized thresholding operator-based methods [25]. For the estimation of sparse low-rank matrices, methods based on penalty terms have also been proposed [28,29]. In addition to that, there is another approach to estimate the covariance matrix based on the modified Cholesky decomposition (MCD) [30]. This covariance matrix estimation certainly has beneficial properties when the number of variables is high. However, the estimate depends on the order of variables [31]. To tackle the problem, several methods have been proposed [32,33,34,35].

The proposed approach adopts an approach that uses hard thresholding based [20,24] therefore, it is easy to use and provides interpretable results. We describe why we aimed to estimate a low-rank sparse correlation matrix rather than a low-rank sparse covariance matrix. The covariance matrix depends on the original multivariate data scale. Since the threshold corresponding to all covariances cannot be treated with a single value, multiple thresholds must be selected by cross-validation. In other words, estimating a low-rank covariance matrix requires multiple threshold-based approaches, which takes an enormous amount of time. On the other hand, estimating a low-rank correlation matrix can be implemented with a single-threshold approach because the scale is uniform, and can be computed relatively quickly. Furthermore, when estimating the covariance matrix, the variance of each variable must also be estimated, which increases the number of parameters to be estimated compared to the estimation of the correlation matrix. Based on the above, this method focuses on the correlation matrix from the feasibility viewpoint and develops a method for estimating sparse low-rank correlation matrices. For more on this discussion, see Refs. [14,36].

The summary of the proposed method is as follows: for small sample sizes and noisy data, a sparse low-rank correlation matrix can be used to emphasize the correlation coefficients between related variables. In addition, the inclusion of sparse constraints eliminates the problem of misunderstanding and facilitates interpretation. Furthermore, the direct estimation of the correlation matrix instead of the covariance matrix reduces the computation time comparatively. We introduce a new cross-validation function to estimate sparse low-rank correlation matrices modifying those used in Refs. [20,24]. This cross-validation function is the mean difference between the low-rank correlation matrix and the original-scale correlation matrix. Compared to the cross-validation function of Refs. [20,24], the value of the proposed cross-validation function tends to be higher when the rank is set to lower. The proposed cross-validation function is expected to choose threshold values corresponding to a more sparse correlation matrix from the property. To calculate the values of the cross-validation function, the majorize-minimization algorithm (MM algorithm) [3,4] and the hard thresholding approach are used. The proposed method has two advantages; first, the estimated sparse low-rank correlation matrix allows an easy and visual interpretation of the correlation matrix using a heatmap and avoids a misinterpretation of the correlation matrix. The proposed approach can estimate the low-rank correlation matrix, and more sparse and specific relations between variables can be emphasized. If the true correlation coefficient is zero, the proposed method estimates the corresponding coefficient as zero. In addition, we focus only on positive correlation coefficients, not negative correlation coefficients. With the focus on only positive relations, it becomes easy to interpret the features of the relations.

The rest of this paper is structured as follows. We explain the model and algorithm in Section 2. Section 3 evaluates the proposed approach and describes the numerical simulation. The results of applying the proposed method to real data are provided in Section 3. Finally, we present our conclusions in Section 4.

## 2. Method

### 2.1. Adaptive Thresholding for Sparse and Low-Rank Correlation Matrix Estimation

In this section, we present the proposed approach for estimating a sparse low-rank correlation matrix. First, an estimation of a low-rank correlation matrix is described based on the MM algorithm [3,4]. Next, the hard thresholding operator and the proposed cross-validation function are described to achieve the sparse low-rank correlation structure.

#### 2.1.1. Optimization Problem of Low-Rank Correlation Matrices

Let $R=\left(r_{ij}\right)\;r_{ij}\in \left[-1,\;1\right]\;\left(i,j=1,2,\cdots ,p\right)$ and $W=\left(w_{ij}\right)\;w_{ij}\in \left\{0,1\right\}\;\left(i,j=1,2,\cdots ,p\right)$ be the correlation matrix between the variables and the binary matrix, respectively, where *p* is the number of variables. Here, $w_{ii}=1\;\left(i=1,2,\cdots ,p\right)$. Given the number of low dimensions d≤p, and the correlation matrix R and the binary matrix W, the optimization problem of estimating a low-rank correlation matrix is defined as follows.

(1) $$\begin{array}{cc} \; & f\left(Y|\;R,W\right)=∥R-W⊙YY^{T}{∥}_{F}^{2}⟶\mathrm{Min}.\\ \mathrm{subject}\;\mathrm{to} & \end{array}$$

(2) $$\begin{array}{cc} \; & ∥y_{j}∥=1\;\mathrm{for}\;\mathrm{all}\;j=1,2,\cdots ,p. \end{array}$$

where $Y={\left(y_{1},y_{2},\cdots ,y_{p}\right)}^{T},y_{j}={\left(y_{j1},y_{j2},\cdots ,y_{jd}\right)}^{T},y_{jo}\in R\;\left(j=1,2,\cdots ,p;\;o=1,2,\cdots ,d\right)$ is the coordinate matrix of variables on dimensions *d*, ⊙ is the Hadamard product, ${∥\cdot ∥}_{F}$ is the Frobenius norm, and ∥·∥ is $L_{2}$ norm. The objective function in Equation (1) was explained by Ref. [37]. From the constraint (2), $Y^{T}Y$ becomes the correlation matrix.

When Y is estimated, W is fixed. In this situation, Y is calculated based on the MM algorithm. The estimation is described in Section 2.1.2. We introduced a modified cross-validation function to determine W and chose W in Section 2.1.3.

#### 2.1.2. Estimation of Low-Rank Correlation Matrices Based on MM Algorithm

The MM algorithm for estimating a low-rank correlation matrix proposed by Ref. [4] is explained (Algorithm 1). To estimate Y in the closed-form under constraint (2), the quadratic optimization problem for Y must be converted to a linear optimization one. We can derive the updated formula combined with the Lagrange multiplier in the closed-form using the linear function. Let $y^{\left(t\right)}\in R^{d}$ be the parameter of the *t* step of the optimization problem algorithm, and $g(y|y^{\left(t\right)})$ be a real function $g:R^{d}\times R^{d}\mapsto R$. If $g(y|y^{\left(t\right)})$ satisfy the following conditions such that

(3) $$\begin{array}{cc} \forall y\in R^{d};\;g\left(y|y^{\left(t\right)}\right)\geq & f(y)\;\;\mathrm{and} \end{array}$$

(4) $$\begin{array}{cc} g(y^{\left(t\right)}|y^{\left(t\right)})= & f(y^{\left(t\right)}), \end{array}$$

$g(y|y^{\left(t\right)})$ is defined as the majorizing function of f(y) at the point $y^{\left(t\right)}$, where $f:R^{d}\mapsto R$ is the original function. Simply put, to estimate the parameters in the MM algorithm, $g(y|y^{\left(t\right)})$, not f(y), should be minimized. In several situations, $g(y|y^{\left(t\right)})$ is expected to easily minimize the value. For more details on the MM algorithm, see Ref. [38].

Before deriving the majorizing function, the objective function (1) can be re-described as follows:

(5) $$\begin{array}{cc} f(Y|\;R,W)= & ∥R-W⊙YY^{T}{∥}_{F}^{2}\\ = & \sum_{i=1}^{p}\sum_{j\neq i}^{p}{\left(r_{ij}-w_{ij}y_{i}^{T}y_{j}\right)}^{2}\\ = & \sum_{i=1}^{p}\sum_{j\neq i}^{p}r_{ij}^{2}+\sum_{i=1}^{p}y_{i}^{T}\left(\sum_{j\neq i}w_{ij}y_{j}y_{j}^{T}\right)y_{i}-2\sum_{i=1}^{p}y_{i}^{T}\left(\sum_{j\neq i}w_{ij}r_{ij}y_{j}\right)\\ = & \sum_{i=1}^{p}\sum_{j\neq i}^{p}r_{ij}^{2}+\sum_{i=1}^{p}y_{i}^{T}B_{i}y_{i}-2\sum_{i=1}^{p}y_{i}^{T}\left(\sum_{j\neq i}w_{ij}r_{ij}y_{j}\right), \end{array}$$

where $B_{i}=\sum_{j\neq i}w_{ij}y_{j}y_{j}^{T}$. Here, the parameter estimation of Y is conducted by $y_{i}$. The corresponding part of Equation (5) and the majorizing function can be described as follows:

(6) $$\begin{array}{cc} f_{i}\left(y_{i}|\;{\left\{y_{j}\right\}}_{j\neq i}\right)= & \;y_{i}^{T}B_{i}y_{i}-2y_{i}^{T}\left(\sum_{j\neq i}w_{ij}r_{ij}y_{j}\right) \end{array}$$

(7) $$\begin{array}{cc} \leq & \;-y_{i}^{(t-1)T}B_{i}y_{i}^{\left(t-1\right)}+2\lambda_{i}-2y_{i}^{T}\left(\lambda_{i}I_{d}-B_{i}\right)y_{i}^{\left(t-1\right)}-2y_{i}^{T}\left(\sum_{j\neq i}w_{ij}r_{ij}y_{j}\right)\\ = & g(y_{i}|\;y_{i}^{\left(t-1\right)},\;{\left\{y_{j}\right\}}_{j\neq i}), \end{array}$$

where $g(y_{i}|\;y_{i}^{\left(t-1\right)},\;{\left\{y_{j}\right\}}_{j\neq i})$ represents the majorizing function of Equation (5), $I_{d}$ is d×d identity matrix, $\lambda_{i}$ is the maximum eigenvalue of $B_{i}$, and $y_{i}^{\left(t-1\right)}$ is $y_{i}$ of (t−1) step in the algorithm. Here, the inequality of Equation (7) is satisfied because $B_{i}-\lambda_{i}I_{d}$ is semi-definitely negative. In fact, if $y_{i}=y_{i}^{\left(t-1\right)}$, Equations (6) and (7) become equal.

Using the Lagrange multiplier method and Equation (7), the updated formula of $y_{i}$ is derived as follows:

(8) $$\begin{array}{c} y_{i}^{\left(t\right)}\leftarrow \frac{\lambda_{i}y_{i}^{\left(t-1\right)}-B_{i}y_{i}^{\left(t-1\right)}+\sum_{j\neq i}w_{ij}r_{ij}y_{j}}{∥\lambda_{i}y_{i}^{\left(t-1\right)}-B_{i}y_{i}^{\left(t-1\right)}+\sum_{j\neq i}w_{ij}r_{ij}y_{j}∥}\;\left(i=1,2,\cdots ,p\right). \end{array}$$

**Algorithm 1** Algorithm for estimating the low-rank correlation matrix**Input:**R, d(≤p) and small constant ε>0**Output:**Y  *Initialisation*: Set $Y^{\left(0\right)}$ satisfying $∥y_{j}^{\left(0\right)}∥=1$ for all *j* and set t←1 1: **while**
$f\left(Y^{\left(t-1\right)}\right)-f\left(Y^{\left(t\right)}\right)\geq \varepsilon$
**do** 2:  **for**
i=1 to *p* 3:    Calculate $B_{i}^{\left(t\right)}\leftarrow \sum_{j\neq i}w_{ij}y_{j}^{\left(t\right)}y_{j}^{(t)T}$ 4:    Calculate $\;\lambda_{i}$ to be the largest eigenvalue of $B_{i}$ 5:    Update $y_{i}$ based on Equation (8). 6:  **End for** 7:  t←t+1 8: **End while** 9: **return**
$Y^{\left(t\right)}$ with the constraint $∥y_{j}^{\left(t\right)}∥=1$ for all *j*


#### 2.1.3. Proposed Algorithm of Cross-Validation to Determine Hard Thresholds

We adopt hard thresholding to estimate the sparse low-rank correlation matrix in the proposed approach. To determine threshold values, we introduce a cross-validation function based on Ref. [20]. The purpose of this approach is quite simple; that is, to determine the threshold values related to sparse estimation by considering the corresponding rank.

Let h(α)∈(−1,1) be a threshold value of sample correlation coefficients corresponding to the α percentile of correlations, where α∈[0,1] is the percentage point. By setting the percentile point α, the corresponding threshold value h(α) is fixed. For a correlation of $r_{ij}\in \left[-1,1\right]$, the function ${𝟙}_{h(\alpha )}\left[r_{ij}\geq h(\alpha )\right]$ is defined as 1 if $r_{ij}\geq h\left(\alpha \right)$, otherwise ${𝟙}_{h(\alpha )}\left[r_{ij}\geq h\left(\alpha \right)\right]=0$. Using them, the proportional threshold operator is defined as

(9) $$\begin{array}{c} T_{h(\alpha )}\left(R\right)=\left({\tilde{r}}_{ij}\right),\;\left(i,j=1,2,\cdots ,p\right) \end{array}$$

where

(10) $$\begin{array}{c} {\tilde{r}}_{ij}=r_{ij}\cdot {𝟙}_{h(\alpha )}\left[r_{ij}\geq h\left(\alpha \right)\right]\;\left(i,j=1,2,\cdots ,p\right). \end{array}$$

For example, the proportional threshold operator is used in the domain of neural science [39]. Let $W_{h(\alpha ),R}=\left(w_{ij}^{\left(h(\alpha )\right)}\right)=\left({𝟙}_{h(\alpha )}\left[r_{ij}\geq h\left(\alpha \right)\right]\right)\in {\left\{0,1\right\}}^{p\times p}$ for correlation matrix R. By using $W_{h(\alpha ),R}$, Equation (9) can be described as follows:

(11) $$\begin{array}{c} T_{h(\alpha )}\left(R\right)=W_{h(\alpha ),R}⊙R. \end{array}$$

Here, Equation (10) is modified for the original function of Ref. [20]. Initially, ${𝟙}_{h(\alpha )}\left[\right|r_{ij}\left|\geq h\left(\alpha \right)\right]$ was used; however, we focused only on higher correlation coefficients and not on negative correlation coefficients. Using modifications, it becomes easy to interpret the results.

**Figure 1:**
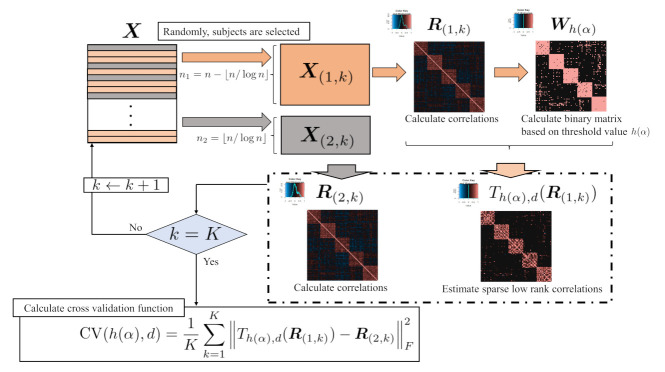
The framework of the proposed cross-validation.

To estimate a sparse low-rank correlation matrix, we introduce the modified proportional threshold operator based on Equation (9) because the interpretation of the proportional threshold is quite simple. Given an α percentile, rank *d*, and the correlation matrix R, the modified threshold operator is defined as follows;

(12) $$\begin{array}{c} T_{h(\alpha ),d}\left(R\right)=W_{h(\alpha ),R}⊙YY^{T} \end{array}$$

where Y is the estimated correlation matrix with rank *d*, such as minimizing $f(Y|\;R,W_{h(\alpha ),R})$. Equation (12) is different from Equation (11) using a low-rank correlation matrix, although $W_{h(\alpha ),R}$ is calculated from the original correlation matrix, not from a low-rank correlation matrix. For the choice of the threshold value h(α), cross-validation was introduced (e.g., Refs. [20,24]). The cross-validation procedure for estimating h(α) consists of three steps, as shown in Figure 1. First, the original multivariate data $X\in R^{n\times p}$ is split into two groups randomly, such as $X_{\left(1,k\right)}\in R^{n_{1}\times p}$ and $X_{\left(2,k\right)}\in R^{n_{2}\times p}$, where $n_{1}=n-\left\lfloor n/\log n\right\rfloor$, $n_{2}=\left\lfloor n/\log n\right\rfloor$, and *k* represent the index of the number of iterations for cross-validation, and ⌊·⌋ represents floor function. For $n_{1}$ and $n_{2}$, Ref. [20] determines both $n_{1}$ and $n_{2}$ from the point of view of theory. Second, the correlation matrices for both $X_{\left(1,k\right)}$ and $X_{\left(2,k\right)}$ are calculated as $R_{\left(1,k\right)}$ and $R_{\left(2,k\right)}$, respectively. Third, the correlation matrix with rank *d*, Y is estimated, such as minimizing $f(Y|\;R_{\left(1,k\right)},W_{h\left(\alpha \right),R_{\left(1,k\right)}})$ with constraint (2). Fourth, for fixed h(α), the procedure from the first step to the third step is repeated *K* times and the proposed cross-validation function is calculated as follows.

(13) $$\begin{array}{c} \mathrm{CV}\left(h\left(\alpha \right),d\right)=\frac{1}{K}\sum_{k=1}^{K}∥T_{h(\alpha ),d}\left(R_{\left(1,k\right)}\right)-R_{\left(2,k\right)}{∥}_{F}^{2} \end{array}$$

where *K* is the number of iterations for the cross-validation. Among the candidates for threshold values, h(α) is selected as the value, such that the expression in Equation (13) is minimized. The algorithm for the cross-validation is presented in Algorithm 2.
**Algorithm 2** Algorithm of Cross-validation for tuning proportional thresholds**Input:** candidates of threshold values ${\left\{h\left(\alpha \right)\right\}}_{\alpha }$, X and d(≤p)**Output:**$\mathrm{CV}(h{\left(\alpha \right)}^{†},d)$  *Initialisation*: Set v=(NA,NA,⋯,NA) with the length of ${|\left\{h\left(\alpha \right)\right\}}_{\alpha }|$  1: **For**
i=1
**to**
${|\left\{h\left(\alpha \right)\right\}}_{\alpha }|$  2:  **For**
k=1 to *K*  3:   Split X into $X_{\left(1,k\right)}$ and $X_{\left(2,k\right)}$  4:   Calculate both $R_{\left(1,k\right)}$ and $R_{\left(2,k\right)}$  5:   Calculate $W_{h\left(\alpha \right),R_{\left(1,k\right)}}$ from corresponding h(α) and $R_{\left(1,k\right)}$.  6:   Given $R_{\left(1,k\right)}$ and $W_{h\left(\alpha \right),R_{\left(1,k\right)}}$, **Algorithm 1** is applied to estimate Y,  7:  **End For**  8:  calculate CV(h(α),d)  9:  v[i]←CV(h(α),d)10: **End For**11: $h{\left(\alpha \right)}^{†}\leftarrow {\mathrm{argmin}\{v\left[1\right],v\left[2\right],\cdots ,v[|\left\{h\left(\alpha \right)\right\}}_{\alpha }\left|]\right\}$12: **return**
$h{\left(\alpha \right)}^{†}$


Finally, $h{\left(\alpha \right)}^{†}$, corresponding to the minimum value of Equation (13) among the candidate threshold values, is selected, and $W_{h{\left(\alpha \right)}^{†},R}⊙YY^{T}$ is estimated based on Equation (1).

### 2.2. Numerical Simulation and Real Example

This section presents a numerical simulation to evaluate the proposed approach. The numerical simulation was conducted based on Ref. [19], with some modifications. Practically, the size of the numerical data is matched to that of the real-data example in Section 2.2.2. In addition, we present a real example of applying the proposed method to a microarray gene expression data set from Ref. [40].

#### 2.2.1. Simulation Design of Numerical Simulation

In this subsection, the simulation design is presented. The framework of the numerical simulation consists of three steps. First, artificial data with a true correlation matrix are generated. Second, sparse low-rank correlation matrices are estimated using two methods, including the proposed method. In addition, a sample correlation matrix and sparse correlation matrix based on the threshold also apply. Third, using several evaluation indices, these estimated correlation matrices are evaluated and their performances are compared.

**Figure 2:**
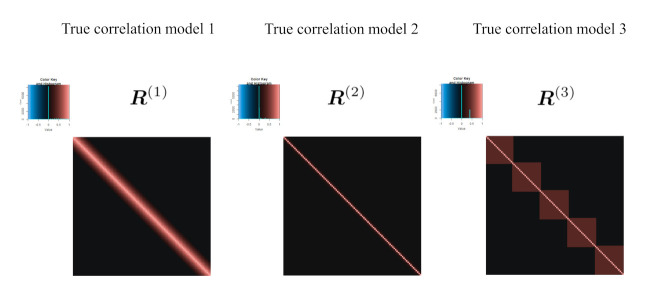
True correlation models.

In this simulation, three kinds of correlation models are used. Let *I* and *J* be a set of indices for the rows and columns of the correlation matrices, respectively. In addition, $I_{k}$ and $J_{k}$ are defined as follows:

$$\begin{array}{cc} I_{k}= & \{i_{(k-1)20+1},i_{(k-1)20+2},\cdots ,i_{(k-1)20+20}\}\;\mathrm{and}\\ J_{k}= & \left\{j_{(k-1)20+1},j_{(k-1)20+2},\cdots ,j_{(k-1)20+20}\right\}\;\left(k=1,2,\cdots ,5\right), \end{array}$$

where $i_{o}$ and $j_{o}\;\left(o=1,2,\cdots ,100\right)$ indicate the number of rows and columns, respectively. Using this notation, three true correlation models, $R^{\left(1\right)}=\left(r_{ij}^{\left(1\right)}\right)$, $R^{\left(2\right)}=\left(r_{ij}^{\left(2\right)}\right)$, and $R^{\left(3\right)}=\left(r_{ij}^{\left(3\right)}\right)$ are set as

(14) $$\begin{array}{cc} r_{ij}^{\left(1\right)}= & {\left(1-\frac{\left|i-j\right|}{10}\right)}_{+}\;\left(i,j=1,2,\cdots ,p\right), \end{array}$$

(15) $$\begin{array}{cc} r_{ij}^{\left(2\right)}= & 0.3^{\left|i-j\right|},\;\left(i,j=1,2,\cdots ,p\right)\;\mathrm{and}, \end{array}$$

(16) $$\begin{array}{cc} r_{ij}^{\left(3\right)}= & 0.6{𝟙}_{i=j}+0.4\sum_{k=1}^{5}{𝟙}_{i\in I_{k},j\in J_{k}}+0.4\sum_{k=1}^{5}\left({𝟙}_{i=i_{k},j\in J_{k+1}}+{𝟙}_{i\in I_{k+1},j=i_{k+1}}\right), \end{array}$$

respectively, where $i_{k}$ and $j_{k}$ are the maximum number of indices of $I_{k}$ and $J_{k}$, respectively, and 𝟙 represents the indicator function. The models for Equations (14) and (15) are called sparse models, while the model for Equation (16) is called a non-sparse model by Ref. [19]. The models for Equations (14) and (15) are used in Refs. [12,13,20]; for these, see Figure 2. These artificial data are generated as $x_{i}∼N\left(0_{p},R^{\left(\ell \right)}\right)\;\left(i=1,2,\cdots ,n;\;\ell =1,2,3\right)$, where $0_{p}$ is a zero vector with a length of *p*. In this simulation, we set p=100 and the number of cross-validations K=5. In this simulation, there are several types of scenarios. For the number of scenarios in the estimation of sparse low-rank correlation matrices, there are 2 (setting 1) × 3 (setting 2) × 3 (setting 3) × 2 (setting 4: proposal and tandem) = 36 patterns. In addition to that, for the number of scenarios in the estimation of sparse correlation matrices without low-rank approximation, there are 2 (setting 1) × 3 (setting 3) × 3 (setting 4: sample correlation, Jiang (2013) with modification and graphical lasso [41]) = 18 patterns. Simply, there are 54 patterns in this numerical simulation. In each pattern, artificial data are generated 100 times and evaluated using several indices. Given W and rank *d*, the result of the estimation Y depends on the initial parameters $Y^{\left(0\right)}$ in both the proposed approach and the tandem approach. Therefore, the low-rank matrix is estimated from 50 randomly generated initial values, and the solution with the smallest value of the objective function is adopted. For $R_{1}$ and $R_{2}$, the candidates of α are set from 0.66 to 0.86 in steps of 0.02. However, for $R^{\left(3\right)}$, the candidates of α are set from 0.66 to 0.82 in steps of 0.02. From the point of view of computation time, we set the range of threshold values. The threshold range is different in the case of $R^{\left(3\right)}$ because above 0.84 it is too sparse to compute the solution of the low-rank matrix.

Next, the settings of the numerical simulation are presented. For the summary, see Table 1. Setting one was set to evaluate the effects of the number of subjects. If the number of subjects is smaller, the estimated sparse low-rank correlation is expected to be unstable. To evaluate the effect of rank, setting two was set. The variance between the estimated sparse low-rank correlation coefficients becomes larger when a smaller rank is set. Therefore, it becomes easy to interpret the results. Next, as explained in Equations (14)–(16), there are three levels in setting three.

Finally, in setting four, we selected five methods: the proposed approach, tandem approach, sample correlation matrix calculation, and the sparse correlation matrix estimation based on threshold value [24] with modifications and graphical lasso [41]. The tandem approach was included in the comparison method to compare the performance of the proposed method, which considers low ranks when computing the cross-validation function, and the tandem approach, which combines existing methods. Jiang (2013) with modifications was included in the comparison method to compare the performance of the proposed method with the case where the low-rank approximation is not considered. Graphical lasso is included as a comparison method because it is often used to infer relationships between variables. However, since the method does not directly estimate the sparse correlation matrix, the correlation matrix is calculated by estimating the inverse of the sparse covariance matrix and then computing the inverse of the covariance matrix. Finally, the sample correlation matrix results are also presented for reference. Next, we explain the compared methods. The purpose of both the proposed and tandem approaches is to estimate a sparse low-rank correlation matrix. In the tandem procedure, there are two steps. The threshold value h(α) is determined based on the following cross-validation function in the first step.

(17) $$\begin{array}{c} \frac{1}{K}\sum_{k=1}^{K}∥W_{h\left(\alpha \right),R_{\left(1,k\right)}}⊙R_{\left(1,k\right)}-R_{\left(2,k\right)}{∥}^{2} \end{array}$$

Equation (17) is a modification of the cross-validation function in Ref. [24]. The modification part is $W_{h(\alpha ),R}=\left(w_{ij}^{\left(h(\alpha )\right)}\right)=\left({𝟙}_{h(\alpha )}\left[r_{ij}\geq h\left(\alpha \right)\right]\right)\in {\left\{0,1\right\}}^{p\times p}$, although $W_{h(\alpha ),R}=\left(w_{ij}^{\left(h(\alpha )\right)}\right)=({𝟙}_{h(\alpha )}\left[\right|r_{ij}|\geq h\left(\alpha \right)])\in {\left\{0,1\right\}}^{p\times p}$ in Ref. [24]. In the second step, using h(α) in the first step, the low rank correlation matrix is estimated based on Ref. [4]. In short, given h(α) and R, Y is estimated as follows:

$$\begin{array}{c} ∥R-W_{h(\alpha ),R}⊙YY^{T}{∥}_{F}^{2}\to \mathrm{Min} \end{array}$$

with the constraint $∥y_{j}∥=1\left(j=1,2,\cdots ,p\right)$. To estimate the sparse correlation matrix without dimensional reduction, there are two methods in this simulation. First, Jiang (2013) with modifications is explained, and this approach is also two steps. The first step is the same procedure as the first step in the tandem approach, and the threshold value h(α) is determined. In the second step, by using h(α) in the first step, the sparse correlation matrix is calculated as $W_{h(\alpha ),R}⊙R$. For the modification, Equation (10) is used as the threshold function (although $r_{ij}\cdot {𝟙}_{h(\alpha )}\left[\right|r_{ij}\left|\geq h\left(\alpha \right)\right]$ was used in Ref. [24]). The second approach is graphical lasso [41]. To select the tuning parameters, the CVglasso package is used [42] in R. The correlation matrix is then calculated from the estimated sparse inverse covariance matrix.

Likewise, as in the approach pursued in Ref. [19], we adopt four evaluation indices. To evaluate the fitting between the estimated sparse low-rank correlation matrix and the true correlation matrix, the average relative errors of the Frobenius norm (F-norm) and the spectral norm (S-norm) are adopted as follows:

(18) $$\begin{array}{cc} \mathrm{F-norm}(\hat{R})= & \frac{∥\hat{R}-R^{\left(\ell \right)}{∥}_{F}}{∥R^{\left(\ell \right)}{∥}_{F}}\;\;\;\mathrm{and} \end{array}$$

(19) $$\begin{array}{cc} \mathrm{S-norm}(\hat{R})= & \frac{∥\hat{R}-R^{\left(\ell \right)}{∥}_{S}}{∥R^{\left(\ell \right)}{∥}_{S}}, \end{array}$$

where ${∥\cdot ∥}_{S}$ indicates the spectral norm, $\hat{R}$ is an estimator of the sparse low-rank correlation matrix, and $R^{\left(\ell \right)}\;\left(\ell =1,2,3\right)$ is the true correlation matrix corresponding to Equation (14), Equation (15), and Equation (16), respectively. In addition, to evaluate the results on sparseness, the true positive rate (TPR) and the false positive rate (FPR) are defined as follows:

(20) $$\begin{array}{cc} \mathrm{TPR}= & \frac{\left|\{\left(i,j\right)|\;{\hat{r}}_{ij}\neq 0,\;r_{ij}^{\left(\ell \right)}\neq 0\}\right|}{\left|\{\left(i,j\right)|\;r_{ij}^{\left(\ell \right)}\neq 0\}\right|}\;\mathrm{and} \end{array}$$

(21) $$\begin{array}{cc} \mathrm{FPR}= & \frac{\left|\{\left(i,j\right)|\;{\hat{r}}_{ij}\neq 0,\;r_{ij}^{\left(\ell \right)}=0\}\right|}{\left|\{\left(i,j\right)|\;r_{ij}^{\left(\ell \right)}=0\}\right|} \end{array}$$

where |·| indicates the cardinality of a set, $\hat{R}=\left({\hat{r}}_{ij}\right)$, and $R^{\left(\ell \right)}=\left(r_{ij}^{\left(\ell \right)}\right)\;\left(\ell =1,2,3\right)$. In addition to that, to evaluate the interpretability, we adopt the following index.

(22) $$\begin{array}{c} \mathrm{Sparsity}=\frac{\left|\{\left(i,j\right)|\;{\hat{r}}_{ij}=0\}\right|}{p\times p-p}. \end{array}$$

Equation (22) ranges from 0 to 1. If Equation (22) is close to 1, the estimated correlation matrix is very sparse, otherwise, that is not sparse.

#### 2.2.2. Application of Microarray Gene Expression Dataset

Here, we present the results of applying both the proposed approach and the tandem approach to the microarray gene expression dataset in Ref. [40]. This real application aims to evaluate the differences between two classes of genes in the results of estimating sparse low-rank correlation matrices. Concretely, in this real example, true correlation coefficients between classes are assumed to be zero, and the FPR of the proposed approach is compared with that of the tandem approach.

In Ref. [25], the same dataset was used to apply their method. Specifically, the data set provided by the R package “MADE4” [43] is used in this example. The data set includes the 64 training samples and the 306 genes. In addition, there are four types of small round blue cell tumors of childhood (SRBCT), such as neuroblastoma (NB), rhabdomyosarcoma(RMS), and Burkitt lymphoma, a subset of non-Hodgkin lymphoma (BL), and the Ewing family of tumors (EWS). Simply, there are four sample classes in this dataset. As in Ref. [25], these genes are classified into two classes: “informative” and “non-informative”, where genes belonging to “informative” have information to discriminate four classes and those belonging to “non-informative” do not.

**Figure 3:**
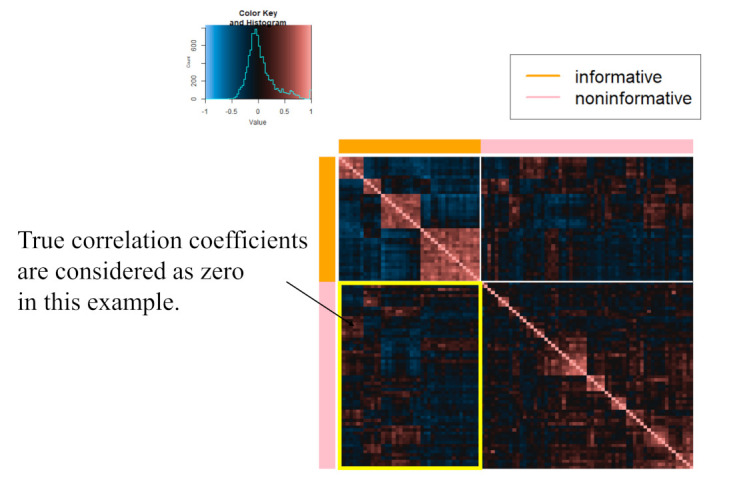
Sample correlation matrix among 100 selected genes.

Next, to construct the “informative” class and the “non-informative” class, the F statistics are calculated for each gene, as follows:

$$\begin{array}{c} F_{j}=\frac{{\left(G-1\right)}^{-1}\sum_{g=1}^{G}n_{g}{\left({\bar{x}}_{gj}-{\bar{x}}_{j}\right)}^{2}}{{\left(n-G\right)}^{-1}\sum_{g=1}^{G}\left(n_{g}-1\right)s_{gj}^{2}}\;\left(j=1,2,\cdots ,306\right) \end{array}$$

where *G* indicates the number of classes, such as NB, RMS, BL, and EWS, $n_{g}$ is the number of subjects belonging to class *g*, ${\bar{x}}_{gj}$ is the mean of class *g* for gene *j*, ${\bar{x}}_{j}$ is the mean of gene *j*, and $s_{gj}$ is the sample variance of class *g* for gene *j*. Here, if $F_{j}$ is relatively higher, gene *j* is considered as “informative” because the corresponding *j* tends to include information such that each class is discriminated. From the calculated $F_{j}$, the top 40 and bottom 60 genes are set as being in the “informative” and “non-informative” classes, respectively. The correlation matrix for 100 genes was then calculated and used as input data. See Figure 3.

To compare the results of the proposed approach with those of the tandem approach, the FPR is calculated. For the tandem approach, see Section 2.2.1. In this application, true correlations between genes belonging to the “informative” class and genes belonging to the “non-informative” class are considered to be 0. Therefore, the FPR denominator is 2×40×60=4800. For TPR, it is difficult to determine the true structure because correlations within each class are not necessarily non-zero. In addition to that, to evaluate the interpretability of the estimated correlation matrix, we adopt the following index:

(23) $$\begin{array}{c} \mathrm{within}\;\mathrm{class}\;\mathrm{Sparsity}=\frac{\left|\{{\hat{r}}_{ij}|\;\left((i,j\in \mathrm{IS})\vee (i,j\in \mathrm{NS})\right),{\hat{r}}_{ij}=0\}\right|}{\left|\{(i,j)|\;i,j\in \mathrm{IS}\}|+|\{(i,j)|\;i,j\in \mathrm{NS}\}\right|} \end{array}$$

where IS and NS are a set of genes belonging to the “informative” and “non-informative” classes, respectively. If Equation (23) is larger, correlation within the same class is considered sparse. For rank, we set 2, 3, and 5. The candidates of α for determining the threshold value are set from 0.50 to 0.83 in steps of 0.01 for both approaches, and these algorithms start from 50 different initial parameters. In addition, as was performed for the numerical simulation, the sample correlation matrix, Jiang (2013) with modifications, and graphical lasso are also employed.

## 3. Results

This section presents the results of the numerical simulation and real application.

### 3.1. Simulation Result

In this subsection, we present the simulation results by the true correlation models. Table 2, Table 3 and Table 4 indicate the FPRs, TPRs, and Sparsity for applying $R^{\left(1\right)}$, $R^{\left(2\right)}$, and $R^{\left(3\right)}$, respectively. Each cell indicates the mean of these indices. Here, $R^{\left(2\right)}$ is a non-sparse correlation matrix and, therefore, FPR cannot be calculated, and both the TPR and FPR of the sample correlation matrix cannot be calculated because the sample correlation is not a sparse matrix. From the results of the numerical simulation, the FPRs of the proposed approach was the lowest among those of all the methods in all the situations, while the TPRs of the proposed approach tended to be inferior to those of the other approaches. Simply, the proposed approach makes it a sparser low-rank correlation matrix compared to the tandem approach when a smaller rank is used. In addition to that, the result of sparsity in the proposed approach was higher than that in the other methods. From the results, we found that the proposed method provides us with interpretable results compared to the tandem method. For the result of the graphical lasso, these correlation matrices are estimated as non-sparse, although these estimated inverse covariance matrices are estimated as sparse. Therefore, both TPRs and FPRs were estimated as higher, and the sparsity in the graphical lasso tends to be 0.00 without the case of $R^{\left(2\right)}$.

**Figure 4:**
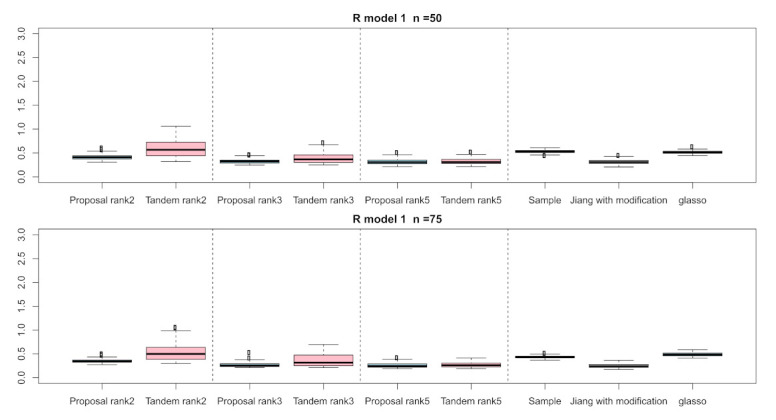
Relative errors of the F-norm for $R^{\left(1\right)}$ with n=50 and n=75; the vertical axis indicates the results of relative errors of the F-norm.

**Figure 5:**
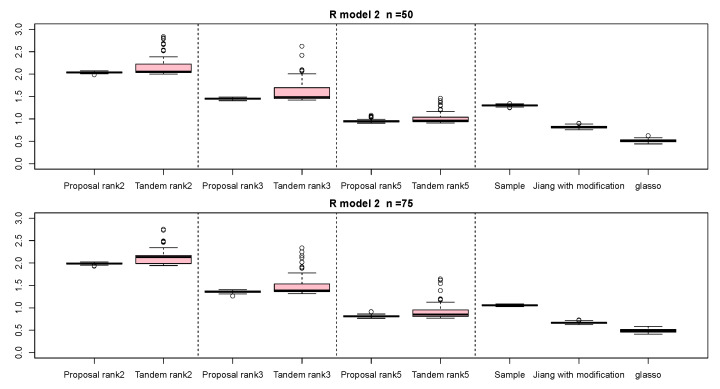
Relative errors of F-norm for $R^{\left(2\right)}$ with n=50 and n=75; the vertical axis indicates the results of relative errors of F-norm.

**Figure 6:**
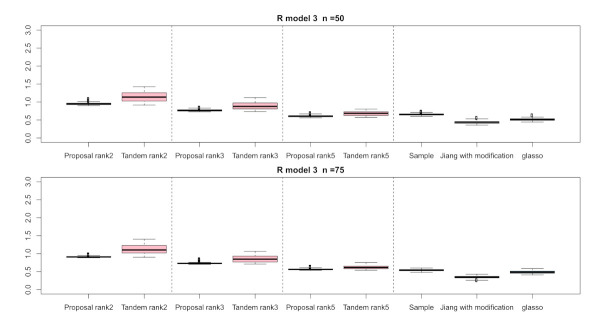
Relative errors of F-norm for $R^{\left(3\right)}$ with n=50 and n=75; the vertical axis indicates the results of relative errors of F-norm.

**Figure 7:**
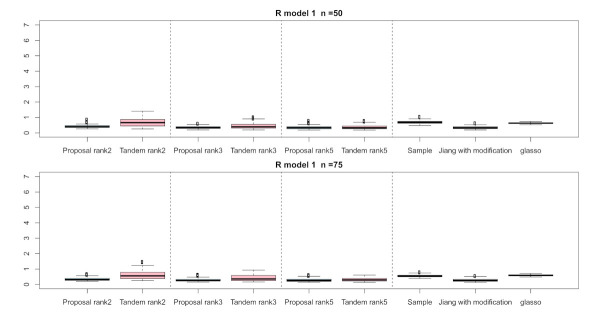
Relative errors of the S-norm for $R^{\left(1\right)}$ with n=50 and n=75; the vertical axis indicates the results of relative errors of the S-norm.

**Figure 8:**
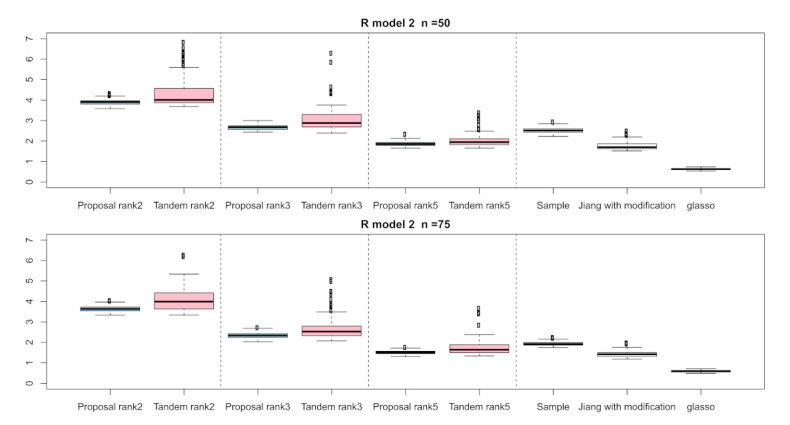
Relative errors of the S-norm for $R^{\left(2\right)}$ with n=50 and n=75; the vertical axis indicates the results of relative errors of the S-norm.

**Figure 9:**
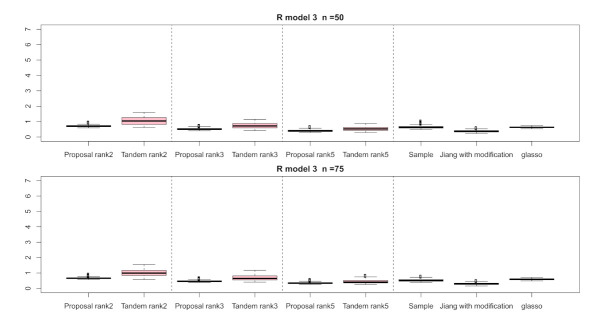
Relative errors of S-norm for $R^{\left(3\right)}$ with n=50 and n=75; the vertical axis indicates the results of relative errors of S-norm.

For the relative error of the F-norm, Figure 4, Figure 5 and Figure 6 indicate the results of applying these methods to $R^{\left(1\right)}$, $R^{\left(2\right)}$, and $R^{\left(3\right)}$, respectively. Hence, the median of the proposed approach was lower than that of the tandem approach for each pattern. Furthermore, the interquartile range of the proposed approach was smaller than that of the tandem approach in each pattern. Therefore, we confirmed that the results of the proposed approach are effective and stable compared to those of the tandem approach. As rank is set as larger, the results of both the proposed approaches become lower and close to those of Jiang (2013) with modifications in all situations. Among these methods, the result of Jiang (2013) with modifications is the best for the relative error of F-norm in the case of $R^{\left(1\right)}$ and $R^{\left(3\right)}$, although that of the graphical lasso (glasso) is the best in the case of $R^{\left(2\right)}$. However, it is a natural thing from the properties of low-rank approximation. As was done for the F-norm, those of S-norm for $R^{\left(1\right)}$, $R^{\left(2\right)}$, and $R^{\left(3\right)}$ are shown in Figure 7, Figure 8 and Figure 9, respectively. The tendency of the results for S-norm is quite similar to that for F-norm. From the results for F-norm, we observe that the result of the proposed approach with rank 5 is quite close to that of Jiang (2013) with modifications.

**Figure 10:**
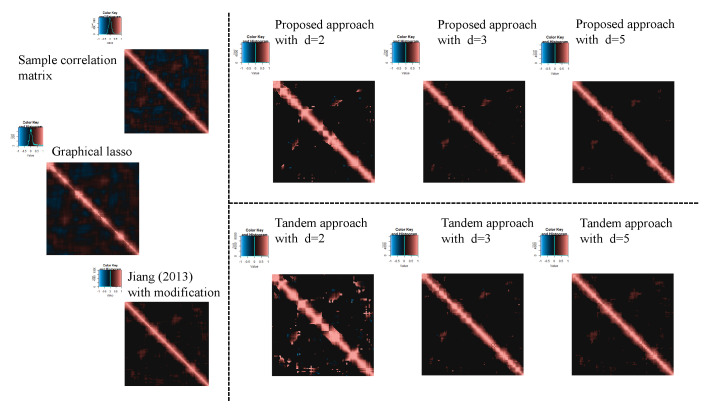
Examples of estimated correlation matrices for true correlation model 1 (n=50).

**Figure 11:**
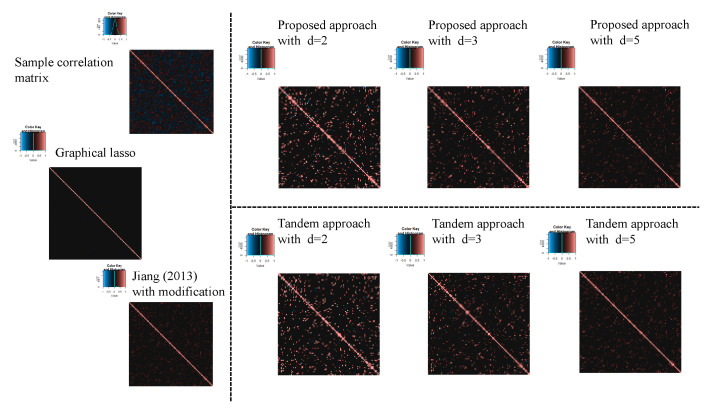
Examples of estimated correlation matrices for the true correlation model 2 (n=50).

**Figure 12:**
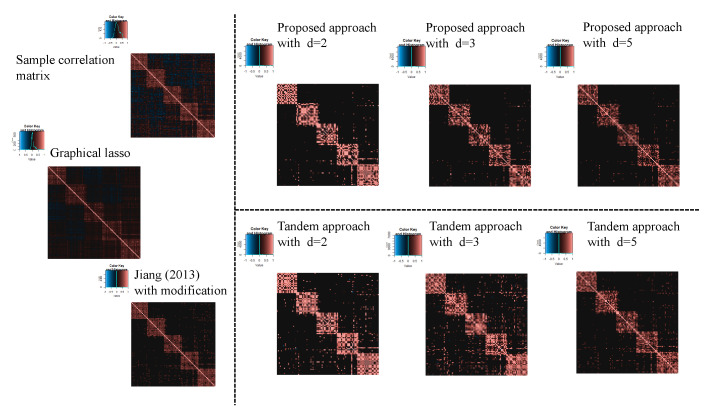
Examples of estimated correlation matrices for the true correlation model 3 (n=50).

**Figure 13:**
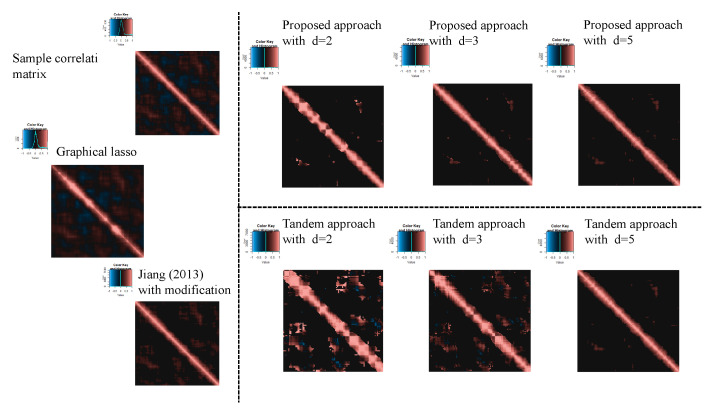
Examples of estimated correlation matrices for the true correlation model 1 (n=75).

**Figure 14:**
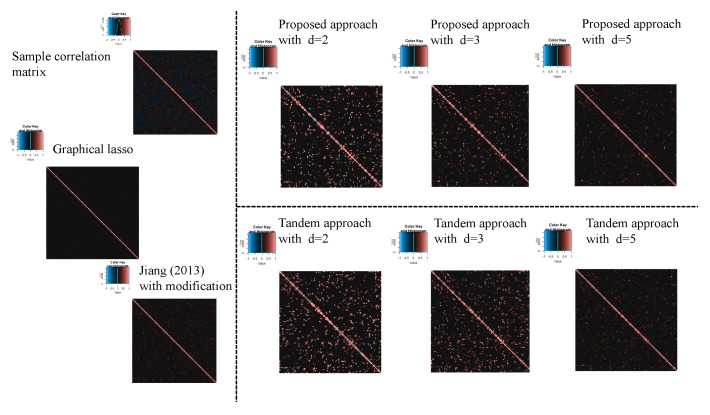
Examples of estimated correlation matrices for the true correlation model 2 (n=75).

**Figure 15:**
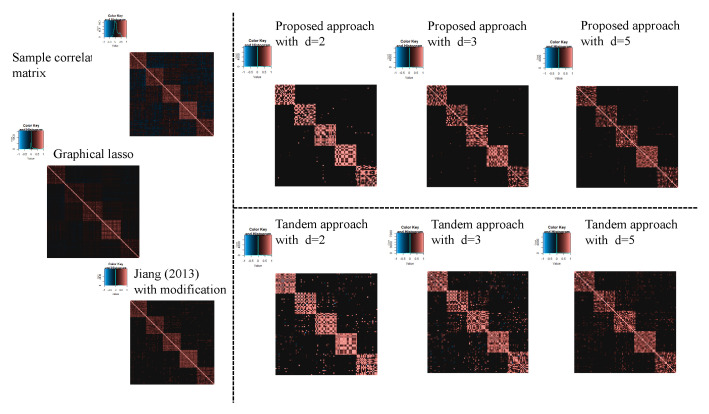
Examples of estimated correlation matrices for true correlation model 3 (n=75).

For the estimated correlation matrices, Figure 10, Figure 11 and Figure 12 correspond to the true correlation model one, the true correlation model two, and the true correlation model three with n=50, respectively. In the same way, Figure 13, Figure 14 and Figure 15 correspond to true correlation model one, true correlation model two, and true correlation model three with n=75, respectively. From Figure 10, Figure 12, Figure 13 and Figure 15, we found that the estimated correlation matrices of the proposed approach tend to estimate zero correctly compared to those of the tandem approach. Especially, the tendency can be confirmed when the rank is set as lower, visually. In addition, rank is set larger, and the estimated correlation matrices tend to be close to the results of Jiang (2013) with modifications.

### 3.2. Result of Application of Microarray Gene Expression Dataset

**Figure 16:**
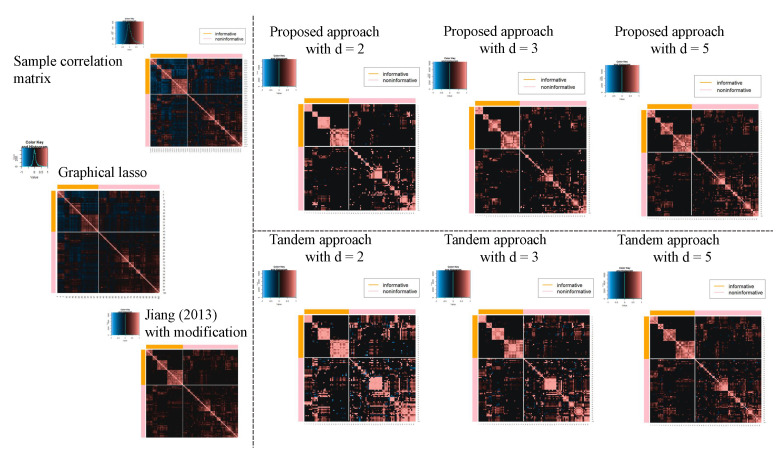
Estimated sparse low-rank correlation matrices with d=2,3, and 5, sample correlation matrix, and sparse correlation matrix without rank reduction.

In this subsection, the results of the application of the microarray gene expression dataset are shown. For the estimated original correlation matrix, Jiang (2013) with modifications, the proposed approach tandem approach, and graphical lasso, see Figure 16. Therefore, the percentage points of d=2,3 and 5 in the proposed approach were estimated as α=0.82,0.81, and 0.75, respectively, while the percentage points in the tandem approach and Jiang (2013) with modifications were both α=0.65. The estimated results of Jiang (2013) with modifications are presented in Figure 16. However, FPRs were higher than those of the proposed approach. Here, the FPR is unaffected by the choice of rank in the tandem approach. From these results, the estimated sparse low-rank correlation matrix tends to be sparser when the rank is set as lower. In fact, it can be confirmed in Figure 16. In addition, as the rank is set larger, the estimated correlations of the proposed approach become similar to those of the tandem approach. We also confirmed that the estimated sparse low-rank correlation matrix between genes belonging to the “informative” class tends to be similar to the results obtained in Ref. [25] using the heatmap.

Next, Table 5 shows the results of the FPR of the proposed approach, the tandem approach, Jiang (2013) with modifications, and the graphical lasso. The FPRs of the proposed method with d=2,3, and 5 were all lower than those of the tandem approach, Jiang (2013) with modifications, and graphical lasso. In addition, the tendency can be confirmed in Figure 16 visually. In fact, we can confirm that the proposed method was able to estimate the correlation coefficient between the classes as zero, compared to the tandem approach. The tendency was observed to be with respect to the results of the numerical simulations.

## 4. Conclusions

This study proposed a novel estimation method for sparse low-rank correlations based on the MM algorithm. The approach overcomes the problem of estimating low-rank correlation matrices. The low-rank approximation is an a potent tool, and the approach provides us with a straightforward interpretation of the features because the contrast of the estimated coefficients becomes larger. However, these estimates sometimes lead to misinterpretations. Even if the true correlation coefficient is zero, the corresponding estimated coefficient of the low-rank approximation without sparse estimation may be greater than zero. To confirm the efficiency of the proposed method, we performed numerical simulations and experimented using a real example, which involved the use of a microarray gene expression dataset. In the case of the real example, the FPR of the proposed approach with d=2,3, and 5 were found to be 0.128, 0.139, and 0.197, respectively, although those of the tandem approach and Jiang (2013) with modifications were found to be 0.285 and 0.285, respectively. We were, therefore able to confirm that the FPR of the proposed approach was the best, irrespective of the rank. Similarly, from the numerical simulations, we confirmed that the FPR of the proposed approach was superior to that of the tandem approach and Jiang (2013) with modifications. In addition to that, we also found that these relative errors of the proposed approach were superior to those of the tandem approach through numerical simulations. The proposed approach is considered an approximate true correlation matrix compared to the tandem approach.

Next, we refer to each method compared. The sample correlation matrix was used as a reference and for comparison. For Jiang (2013), concerning modifications, the numerical simulation results confirm that the F-norm and the S-norm are better than the proposed approach and the tandem approach because the low-rank approximation is not performed. Furthermore, Jiang (2013) with modifications is better than the sample correlation matrix without sparse constraints. Graphical lasso is a method for the sparse estimation of the inverse of the covariance, and the computed correlation matrix is not necessarily a sparse. Therefore, FPR, sparsity, and within-class sparsity were not good compared to the other methods through numerical simulations and examples of real-data applications. In other words, when a correlation matrix is estimated, it would be better to estimate a direct correlation matrix or a covariance matrix. The TPR, FPR, and sparsity of the tandem approach are almost identical to Jiang (2013) with modifications. The tandem method has a higher FPR than the proposed method and worse results for sparsity and within-class sparsity, which are indicators of interpretability. Furthermore, the proposed method performed better for the F-norm and the S-norm because the tandem method does not take threshold values into account. However, the numerical simulation results show that the TPR tends to be better than the proposed method.

Here, we show the recommendations of the proposed approach. First, the proposed approach can provide the interpretable sparse low-rank correlation matrix from the result of both the sparsity of the numerical simulation and the within-class sparsity of the real example. Second, in a real example, the sparse low-rank correlation matrix estimated by the proposed approach was close to previous studies [21]. However, additional constraint, such as a low-rank approximation, is added to the proposed method. Third, the proposed approach can reduce the FPR compared to the tandem approach and the graphical lasso in this study. In short, the proposed approach can avoid misunderstandings compared to those methods.

Although the proposed approach showed promising results in both experiments, several limitations need further investigation. First, when the rank is set low, the TPRs of the proposed method were low compared to those of the tandem approach. The relationship between the determination of the rank and the corresponding TPR, F-norm, S-norm, sparsity, and within-class sparsity was a trade-off. Therefore, a method for a more effective determination of the rank should be developed. As one of the solutions to the problem, we can consider the introduction of a nuclear-norm regularization [44]. Second, when the percentage point is set significantly higher, it may be challenging to obtain the sparse low-rank correlation matrix because it becomes difficult to calculate an updated formula of the low-rank correlation matrix. Third, the proposed approach was more sparse than the tandem approach through numerical simulations and real examples, but this was not shown theoretically. This point also needs to be studied theoretically, but this is a topic for future research. Finally, it should be noted that the proposed approach only focuses on the positive correlation matrix and does not consider the negative correlation matrix for the simplicity of the interpretation. However, the proposed method can be extended for considering both positive and negative correlation coefficients.

## Figures and Tables

**Table 1:** Settings of numerical simulation.

Setting Name	Levels	Description
Setting 1: The number of subjects	2	n=50,75
Setting 2: Rank	3	d=2,3,5
Setting 3: True correlation model	3	Equations (14)–(16)
Setting 4: Methods	5	proposed approach, tandem approach, sample correlation, Jiang (2013) with modification, and graphical lasso

**Table 2:** Results of FPRs and TPRs for $R^{\left(1\right)}$. Each value indicates the mean.

p|n	Methods	*d*	FPR	TPR	Sparsity
100|50	Sample correlation matrix		−	−	−
	Jiang (2013) with modifications		0.07	0.82	0.79
	Graphical lasso		1.00	1.00	0.00
	Tandem	2	0.07	0.82	0.79
	Proposal		0.02	0.76	0.85
	Tandem	3	0.07	0.82	0.80
	Proposal		0.03	0.78	0.84
	Tandem	5	0.08	0.82	0.79
	Proposal		0.06	0.81	0.81
100|75	Sample correlation matrix		−	−	−
	Jiang (2013) with modifications		0.06	0.85	0.80
	Graphical lasso		1.00	1.00	0.00
	Tandem	2	0.06	0.85	0.80
	Proposal		0.01	0.79	0.85
	Tandem	3	0.07	0.85	0.79
	Proposal		0.02	0.81	0.84
	Tandem	5	0.07	0.85	0.79
	Proposal		0.05	0.84	0.81

**Table 3:** Results of FPRs and TPRs for $R^{\left(2\right)}$. Each value indicates the mean.

p|n	Methods	*d*	FPR	TPR	Sparsity
100|50	Sample correlation matrix		−	−	−
	Jiang (2013) with modifications		−	0.17	0.84
	Graphical lasso		−	0.47	0.52
	Tandem	2	−	0.17	0.84
	Proposal		−	0.15	0.86
	Tandem	3	−	0.17	0.83
	Proposal		−	0.15	0.86
	Tandem	5	−	0.16	0.84
	Proposal		−	0.15	0.85
100|75	Sample correlation matrix		−	−	−
	Jiang (2013) with modifications		−	0.17	0.84
	Graphical lasso		−	0.97	0.02
	Tandem	2	−	0.17	0.84
	Proposal		−	0.15	0.86
	Tandem	3	−	0.17	0.83
	Proposal		−	0.15	0.86
	Tandem	5	−	0.17	0.83
	Proposal		−	0.15	0.85

**Table 4:** Results of FPRs and TPRs for $R^{\left(3\right)}$. Each value indicates the mean.

p|n	Methods	*d*	FPR	TPR	Sparsity
100|50	Sample correlation matrix		−	−	−
	Jiang (2013) with modifications		0.08	0.93	0.74
	Graphical lasso		1.00	1.00	0.00
	Tandem	2	0.08	0.93	0.74
	Proposal		0.02	0.83	0.81
	Tandem	3	0.08	0.93	0.74
	Proposal		0.02	0.84	0.81
	Tandem	5	0.09	0.94	0.73
	Proposal		0.03	0.86	0.80
100|75	Sample correlation matrix		−	−	−
	Jiang (2013) with modifications		0.07	0.97	0.73
	Graphical lasso		1.00	1.00	0.00
	Tandem	2	0.07	0.97	0.73
	Proposal		0.01	0.86	0.81
	Tandem	3	0.07	0.97	0.74
	Proposal		0.01	0.87	0.81
	Tandem	5	0.07	0.97	0.74
	Proposal		0.02	0.90	0.80

**Table 5:** Results of applying the microarray gene expression dataset.

Method	*d*	FPR	Within Class Sparsity
Proposal	2	**0.128**	0.757
Proposal	3	0.139	0.748
Proposal	5	0.197	0.687
Tandem	−	0.285	0.578
Jiang (2013) with modifications	−	0.285	0.578
Graphical lasso	−	1.000	0.000

## Data Availability

The gene expression data set belongs to the package R ‘MADE4’ in: https://www.bioconductor.org/packages/release/bioc/html/made4.html (accessed on 17 April 2022).

## Abbreviations

The following abbreviations are used in this manuscript:

MM	Majorize-minimization
DOAJ	Directory of open access journals
TLA	Three letter acronym
LD	Linear dichroism
FPR	False Positive Rate
TPR	True Positive Rate
F-norm	Frobenius norm
S-norm	Spectral norm
SRBCT	Small round blue cell tumours of childhood
NB	Neuroblastoma
RMS	Rhabdomyosarcoma
BL	Burkitt lymphoma, a subset of non-Hodgkin lymphoma
EWS	Ewing family of tumours
